# Supplementary material for: Association between oral microbiome and seven types of cancers in East Asian population: a two-sample Mendelian randomization analysis
Source: Front Mol Biosci. 2023 Nov 21;10:1327893. doi: 10.3389/fmolb.2023.1327893 (PMC10702768; doi:10.3389/fmolb.2023.1327893)
Supplement: Supplementary file 1 [file DataSheet1.ZIP › Supplementary Materials/MR plots of saliva/saliva═╝/Gastric cancer/pheno.973_to_gastric cancer_leave_one_out.pdf]

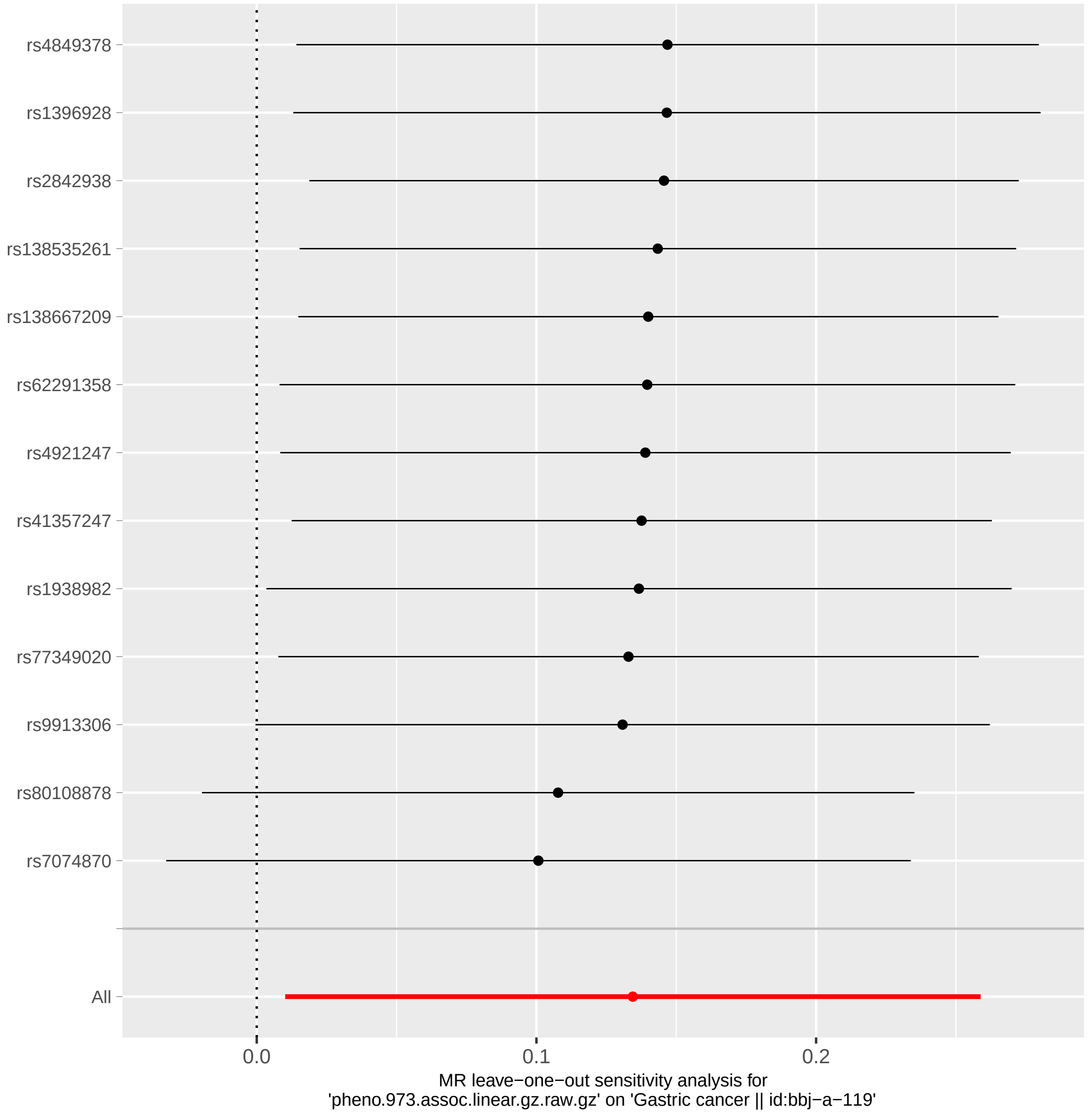
rs4849378
rs1396928
rs2842938
rs138535261
rs138667209
rs62291358
rs4921247
rs41357247
rs1938982
rs77349020
rs9913306
rs80108878
rs7074870
All
0.0
0.1
0.2
MR leave−one−out sensitivity analysis for
'pheno.973.assoc.linear.gz.raw.gz' on 'Gastric cancer || id:bbj−a−119'
